# Interplay between B vitamins, fiber, and *Bacteroides* abundance: a predictive model for anxiety and depression in amyotrophic lateral sclerosis

**DOI:** 10.3389/fmicb.2026.1815390

**Published:** 2026-06-08

**Authors:** Claudia Emmanuela Sanchis-Sanchis, David Sancho-Cantus, Elena Sanchis-Sanchis, Jesús Privado, Francisco J. Roig, María Cuerda-Ballester, Guillermo Bargues-Navarro, Laura Cubero-Plazas, Palmira Martínez Bolós, Ruth Gabriela Cárdenas Salazar, José Enrique de la Rubia Ortí

**Affiliations:** 1 Doctoral School, Catholic University of Valencia San Vicente Mártir, Valencia, Spain; 2 Faculty of Medicine and Health Sciences, Catholic University of Valencia San Vicente Mártir, Valencia, Spain; 3 Department of Methodology of Behavioral Sciences, Universidad Complutense de Madrid, Campus de Somosaguas, Madrid, Spain; 4 Faculty of Health Sciences, San Jorge University, Zaragoza, Spain; 5 BIAMICS, Bioinformatics Services Ltd., Zaragoza, Spain

**Keywords:** amyotrophic lateral sclerosis, anxiety, depression, gut microbiota, vitamin B complex

## Abstract

**Background:**

Amyotrophic lateral sclerosis (ALS) is a progressive and incurable neurodegenerative disease that not only affects motor function but is also associated with gastrointestinal and emotional disturbances. Recent research highlights the potential role of gut microbiota and diet in modulating these symptoms, suggesting a complex interaction between nutrition, intestinal health, and presence of anxiety and depression in ALS patients. This study aims to investigate the relationship between dietary intake, gut microbiota composition, and presence of anxiety and depression in patients with amyotrophic lateral sclerosis (ALS).

**Methodology:**

A cross-sectional study conducted with a sample of 48 patients with bulbar-onset or spinal-onset ALS from different regions of Spain. Dietary intake was assessed through 24-h records and food frequency questionnaires, while anxiety and depression were evaluated using validated scales that formed a latent factor called emotional distress. Stool consistency was assessed following the Bristol Stool Scale and the abundance of bacterial microbiota was quantified.

**Results:**

Confirmatory factor analysis identified a nutritional factor composed of vitamins B1, B2, B9, C, and fiber, revealing a significant inverse association with anxiety and depression levels. The predictive model revealed both direct and indirect effects of this factor on presence of anxiety and depression, mediated by *Bacteroides* abundance and stool consistency.

**Conclusion:**

This model explained 19% of the variance in psychological distress. Our findings suggest that a diet rich in B vitamins, C vitamin and fiber may help improve emotional well-being in patients with ALS, highlighting the importance of nutritional strategies, as well as the role of *Bacteroides* related to stool consistency in patients with ALS.

## 1 Introduction

Amyotrophic lateral sclerosis (ALS) is a rare neurodegenerative disease characterized by the progressive death of motor neurons (Goncharova et al., 2021). As there is currently no cure, improving quality of life through symptom management is paramount (Hobson and McDermott, 2016). The incidence in Spain is 1.4 cases per 100,000 inhabitants per year, with a prevalence of 8–9 cases per 100,000 inhabitants per year and an average survival of 2–5 years from symptom onset (Instituto Nacional de Estadística (INE), 2025).

Clinically, ALS can be classified according to whether symptom onset occurs at the bulbar or spinal level (Ravits and La Spada, 2009). While motor symptoms, including muscle atrophy and fasciculations, are predominant (Ravits and La Spada, 2009), patients with ALS commonly experience gastrointestinal symptoms, including constipation, diarrhea, gas, and abdominal bloating (Hirayama et al., 2023; Nguyen, 2024). Concomitantlly, emotional disorders, particularly anxiety and depression, are prevalent in this population and significantly impact disease progression and quality of life (Thakore and Pioro, 2016).

Emerging evidence suggests that emotional disturbances in ALS may be linked to gut microbiota alterations (Singh et al., 2024), phenomena consistently observed in patients with this condition (Sharma, 2025). Given that gut microbiota composition is directly influenced by dietary patterns (Cuffaro et al., 2024), the nutritional dysregulation commonly observed in patients with ALS (Gibiino et al., 2021) may contribute to both microbiota imbalances and gastrointestinal symptoms (Parra-Cantu et al., 2021). Of particular note are changes in stool consistency, primarily resulting from constipation (hard stools) or, less frequently, episodes of diarrhea, reflecting underlying motility deficits (Gotkine et al., 2020). Furthermore, this complex interplay between nutrition, gut health, and microbiota composition may ultimately influence emotional well-being via the gut-brain axis (Avolio et al., 2019). Mechanistically, this relationship may be explained by the bidirectional communication system of the gut–brain axis, through which gut microbiota can influence central nervous system function via immune, metabolic, and neural pathways. Microbiota-derived metabolites and neurotransmitter precursors have been shown to modulate neuroinflammation, intestinal permeability, and brain signaling processes, which may be particularly relevant in the context of ALS (Cryan et al., 2019).

This study aims to analyze the complex interplay between micronutrient intake, gut microbiota composition, stool consistency, and psychological distress (anxiety and depression) in patients with ALS. We initially examined the factor structure of micronutrient intake to identify a latent factor representing their common variance. Subsequently, we developed a predictive model to evaluate the extent to which specific gut bacteria and stool consistency mediate the effects of this nutritional factor on levels of anxiety and depression within this population.

## 2 Materials and methods

### 2.1 Study design and population

This was a selective cross-sectional study in which participants were recruited based on characteristics relevant to the study objectives. Patients with either bulbar- or spinal-onset ALS from different regions of Spain were included. The sample was identified through the main ALS patient associations in the country, which were first informed about the study objectives. Interested patients received detailed information about the purpose, tests, and analyses to be performed and provided written informed consent.

The following inclusion criteria were applied: men over 18 years of age; female patients over 50 years of age who were infertile, or between 18 and 50 years of age who did not plan to become pregnant (to minimize hormonal confounding); confirmed diagnosis of ALS with a duration of at least six months (to reduce the risk of diagnostic errors in the initial phases and ensure the clinical stability of the participants); active treatment with riluzole as the only drug accepted worldwide for the disease; and provision of written informed consent, in compliance with the ethical principles established in the Declaration of Helsinki.

Exclusion criteria included: patients with tracheostomy; those with invasive or non-invasive positive pressure ventilation; participants in other clinical trials in the previous four weeks; individuals with signs of dementia; alcohol or drug dependence; hepatitis B, C or HIV infections; renal failure (creatinine twice the upper limit of normal in the previous 30 days); liver impairment (ALT or AST levels three times above the upper limit of normal in the previous 30 days); or any diagnosed inflammatory bowel disease or gastrointestinal disease.

Finally, 48 patients with ALS were evaluated, with a mean age of 56.94 years (SD = 10.05 years). Of these, 64.6% were male. 14.6% presented with bulbar ALS and 85.4% with spinal ALS. The mean time since diagnosis was 28.71 months (SD = 27.59 months), ranging from 2 to 146 months.

### 2.2 Measures

#### 2.2.1 Psychological distress

The ALS Depression Inventory-12 (ADI-12) is a 12 item instrument designed to assess depressive symptoms in individuals diagnosed with ALS. The assessment focuses on the mood experienced during the two weeks prior to the interview and uses a four-point Likert-type scale, ranging from “Strongly agree” to “Strongly disagree” (Kübler et al., 2005). In our sample, the internal consistency (Cronbach’s alpha) was 0.920, indicating excellent reliability (Hair et al., 1999).

The Beck Anxiety Inventory (BAI) consists of 21 items designed to assess the intensity of anxiety symptoms through self-report. Each item corresponds to a specific anxiety symptom, and participants indicate the degree to which they experienced it during the previous week, using a four-point Likert scale ranging from “Not at all” to “A lot or I could hardly stand it” (Beck et al., 1988; Sanz et al., 2012). Cronbach’s alpha was 0.909 for the somatic scale, 0.922 for the affective-cognitive scale, and 0.952 for the total scale.

#### 2.2.2 DNA extraction, sequencing, and quality control

Participants collected fecal samples at home within 24 h prior to their hospital visit using the OMNIgene-GUT kit (OMR-200; DNA Genotek, Ottawa, Canada), following detailed instructions for proper collection and storage. Participants transferred a portion of their stool to the kit container, which does not require preservatives. Microbial genomic DNA extraction was performed within one month of collection employing the DNeasy PowerSoil Pro kit (Qiagen, Hilden, Germany), strictly following the manufacturer specifications.

Sequencing was performed via shotgun metagenomics on the Illumina NovaSeq6000 platform with an S4 flow cell (2 × 150 bp). Library preparation was conducted using the Illumina DNA Prep kit. Demultiplexing was performed using bcl2fastq (v2.20.0.422) (Blanco-Míguez et al., 2023), allowing a maximum of one mismatch in barcodes. Read preprocessing included the removal of low-quality sequences (Q < 20), short reads (<75 bp), and those with more than two ambiguous nucleotides, using TrimGalore (Krueger et al., 2016). Contaminating DNA was detected and removed using Bowtie2 (Langmead and Salzberg, 2012), employing the -sensitive-local parameter to filter sequences corresponding to Illumina’s phi X 174 control and the human genome (hg19).

Standardized files of direct, reverse, and unpaired reads were obtained for each metagenome. The generated data are available in a public repository under accession number PRJEB90512.

#### 2.2.3 Taxonomic profile

Taxonomic characterization of microbial communities was performed using MetaPhlAn4 with the mpa_vJan21_CHOCOPhlAnSGB_202103 database^1^ under default parameters. MetaPhlAn4 uses high-quality mapped reads to estimate the coverage of specific marker genes and to calculate relative abundance at different taxonomic levels (Blanco-Míguez et al., 2023; Krueger et al., 2016).

#### 2.2.4 Assessment of stool quantity and consistency

The Bristol Stool Scale. The Bristol scale (Lewis and Heaton, 1997; Parés et al., 2009) allows for stool assessment in a graphic and descriptive manner. Using visual representations, patients classify stools into seven types based on consistency and shape. It is currently the only scale that describes stool shape and is endorsed by consensus groups for data collection in pathological conditions involving functional bowel disorders (Longstreth et al., 2006).

According to the Bristol scale, stools can be classified by transit time as: slow transit (types 1 and 2), normal transit (types 3, 4, and 5), and fast transit (types 6 and 7) (Mínguez and Benages, 2009). For statistical analysis, the scale was treated as an ordinal variable codified from 1 to 7, where lower values indicate harder stools (slower transit) and higher values indicate softer or liquid stools (faster transit).

To achieve a measure ranging from the least desirable to the optimal stool type, the Bristol scale was recoded as follows: 1, severe constipation or diarrhea; 2, constipation or diarrhea; 3, lack of fiber; 4, normal; and 5, optimal. Higher scores indicate better stool consistency.

In accordance with the weekly dietary monitoring, participants reported their stool type using the Bristol scale (Lewis and Heaton, 1997). Information was also collected on bowel movement frequency (per day or week) and associated symptoms including gas, diarrhea, constipation and abdominal pain. Patients also reported any medications or home remedies used to alleviate symptoms.

#### 2.2.5 Nutritional analysis

##### 2.2.5.1 Dietary history and data collection

To assess food intake, 7-day dietary records were used along with a Food Frequency Questionnaire. Both instruments provided detailed information on the consumption frequency of various food groups, including dairy products, vegetables, fruits, juices, nuts, meats, fish, seafood, eggs, tubers, rice, legumes, pasta, processed meats, snacks, pastries, cookies, chocolates, sugary drinks, non-distilled alcoholic beverages, and distilled alcohol. This one-week period provided a representative overview of the participants’ usual eating habits, reducing bias associated with analyzing a single day (Ortega et al., 2015).

The 24-h record consisted of a self-completed questionnaire (Trinidad et al., 2008) in which patients recorded the foods consumed each day and ingredients used in meal preparation. They indicated amounts consumed using common household measures (cup, serving, glass, tablespoon, slice, handful, plate, or ladle) or exact weights. Participants received a guide containing common weight and measurement equivalents (Dapcich et al., 2004). The Food Frequency Questionnaire (Trinidad et al., 2008) included a table for indicating the usual consumption frequency of each food group.

##### 2.2.5.2 Assessment of diet and eating habits

Based on the 7-day dietary record and Food Frequency Questionnaire data, diet quality was assessed using DietoPro® software^2^, a nutritional management tool developed in Valencia, Spain. This software generated individualized nutritional profiles by calculating daily averages of macronutrient and micronutrient intake.

For macronutrients, the software provided data on total energy intake, proteins, carbohydrates, total fats, lipid profile breakdown (monounsaturated, saturated, and polyunsaturated fatty acids), cholesterol, and the percentage distribution of protein, lipids, and carbohydrates.

For micronutrients, daily levels were calculated for sodium, fiber, ethanol, iodine, potassium, calcium, magnesium, phosphorus, iron, selenium, zinc, vitamins B1, B2, B6, and B12, folate, niacin, and vitamins C, A, D. Additionally, composite nutritional indicators were analyzed, such as calcium-to-phosphorus, vitamin E-to-polyunsaturated fatty acids, and vitamin B6-to-protein ratios.

To determine whether participants’ diets met nutritional recommendations, Dietary Reference Intakes (DRIs) were used in accordance with the “Reference Intakes for the Spanish Population” (Cuervo et al., 2010) and the guidelines from the “Consensus of the Spanish Society of Community Nutrition (SENC)” (Aranceta and Serra-Majem, 2011).

### 2.3 Data analysis

First, different descriptive statistics (mean and standard deviation) were calculated for all measurements. Second, Pearson correlations between the variables were computed using SPSS V.23.

Third, a confirmatory factor analysis (CFA) was performed to examine whether vitamin and fiber measurements grouped into a latent factor reflecting their common variance.

Fourth, a predictive structural model was estimated to examine the predictive impact of vitamins, fiber, *Bacteroides* abundance, and stool consistency on psychological distress (anxiety and depression). The specific inclusion of these bacteria in the predictive model was based on the fact that, among the 76 microbiota measures collected, *Bacteroides* was the only genus that exhibited a correlation of at least | ± 0.10| with both anxiety and depression. This criterion was established because, according to Cohen (1992), a correlation value of 0.10 represents a threshold for a small effect size. This confirmatory analysis was performed using AMOS V. 23 (Arbuckle, 2006). Three types of goodness-of-fit indices were used to assess model fit: (1) Absolute fit indices: the χ2/df ratio (Bentler and Bonett, 1980), with values ≤ 3.0 indicating acceptable fit; the Goodness-of-Fit Index (GFI) (Bentler and Bonett, 1980), with values ≥ 0.95 indicating good fit; the Standardized Root Mean Square Residual (SRMR) (Jöreskog and Sörbom, 1993), with values ≤ 0.08 indicating good fit [33], and the presence of < 5.0% of residuals greater than 2.58 in absolute value (Arbuckle, 2006; Hu and Bentler, 1999); (2) Incremental fit indices: the Normed Fit Index (NFI) (Jöreskog and Sörbom, 1993), with values > 0.95 indicating good fit; and 3) Parsimony indices: the Parsimony Goodness of Fit Index (PGFI) (Hair et al., 1999) and the Parsimony Normalized Fit Index (PNFI) (James et al., 1982), with values > 0.50 indicating acceptable fit.

For confirmatory models, 10 participants per indicator are generally recommended for factor analyses (Byrne, 2001), although some authors suggest 5 per indicator when the distribution is normal (Hu and Bentler, 1999). Our study met the latter criterion, with 48 participants for 5–10 indicators across the different models tested (48/5 = 9.60; 48/10 = 4.80).

### 2.4 Ethical considerations

This research was conducted in accordance with the principles of the Declaration of Helsinki and Good Clinical Practice guidelines (World Medical Association, 2013). The study protocol was approved by the Clinical Research Ethics Committee of La Fe Hospital in Valencia, Spain (protocol code 2021–001989-38). All participants provided written informed consent after receiving detailed information regarding the study objectives, procedures, potential risks, and benefits. Participants were informed of their right to withdraw from the study at any time without prejudice to their clinical care.

## 3 Results

### 3.1 Descriptives and correlations

Supplementary Table 1 presents the descriptive statistics (means and standard deviations) for all measures collected in patients with ALS, along with Pearson correlations among the study variables. According to Cohen (1992), effect sizes for correlations are considered small (r = 0.10), medium (r = 0.30), and large (r = 0.60). The variables most strongly correlated with psychological distress (depression and anxiety) included: fiber (r = −0.08 to −0.27), iodine (r = −0.24 to −0.31), ethanol (r = −0.14 to −0.20), magnesium (r = −0.17 to −0.23), vitamin B6 (r = −0.22 to −0.27), vitamin B12 (r = −0.04 to −0.20), vitamin B9 (r = −0.33 to −0.35), vitamin C (r = −0.28 to −0.37), vitamin A (r = −0.24 to −0.29), vitamin E (r = −0.03 to −0.20), stool consistency (r = −0.04 to −0.23), and *Bacteroides* abundance (r = 0.11 to 0.22).

These results indicate that vitamin intake showed significant negative correlations with psychological distress. Specifically, higher intake of the identified vitamins (B6, B9, C, A, and E) was associated with lower levels of psychological distress among the participants. Additionally, other nutrients such as fiber and iodine exhibited negative correlations with psychological distress.

### 3.2 Confirmatory factor analysis

**Figure 1:**
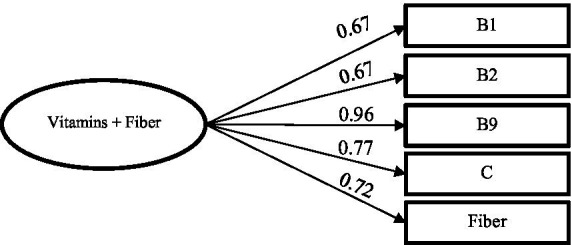
Confirmatory factor analysis for vitamins and fiber.

Based on the correlations between the different measures and psychological distress (anxiety and depression), a confirmatory factor model was estimated to capture the common variance among the various nutrients. Following established criteria (Hair et al., 1999), only variables with factor loadings of at least 0.40 were retained to ensure a substantial contribution to the common factor, the final measures comprising this factor are shown in Figure 1. Other nutrients did not meet the inclusion criteria.

The model was estimated using the Unweighted Least Squares procedure, as the variables included in the model did not exhibit multivariate normality based on the Bolle-Stine bootstrap (Bollen and Stine, 1993) (*p* < 0.005). The goodness-of-fit indices reflect a moderate fit to the data (see Table 1), and the latent factor indicators yielded values above the recommended minimum | ± 0.40| (Hair et al., 1999), ranging specifically from 0.67 to 0.96. These results indicate that the four vitamins included in the model (B1, B2, B9, and C) and fiber share a considerable amount of common variance, allowing them to be grouped into a latent factor termed “Vitamins + Fiber.” This factor accounts for 71.46% of the total variance of the five indicators.

**Table 1:** Goodness-of-fit indices for confirmatory factor analysis and the predictive model.

Model	*χ^2^/df*	GFI	NFI	PGFI	PNFI	SRMR	Residues ≥ | ± 2.58|
Confirmatory factor analysis	85663297.89	0.989	0.975	0.330	0.487	0.231	0.00%
Predictive model	12855965.48	0.989	0.874	0.611	0.756	0.164	0.00%

GFI, Goodness-of-Fit Index; NFI, Normed Fit Index; PGFI, Parsimony Goodness of Fit Index; PNFI, Parsimony Normalized of Fit Index; SRMR, Standardized Root Mean Square.

### 3.3 Predictive model

**Figure 2:**
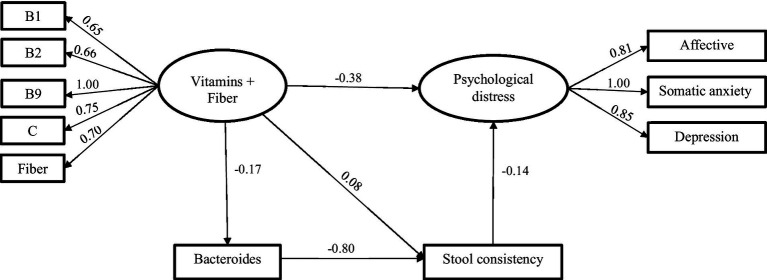
Predictive structural model examining the relationships between micronutrient intake, gut microbiota composition, stool consistency, and psychological distress in patients with ALS. The latent variable Vitamins + Fiber represents the shared variance of dietary intake of vitamins B1, B2, B9, vitamin C, and fiber. Psychological distress is a composite measure derived from anxiety (BAI) and depression (ADI-12) scores. Bacteroides abundance refers to the relative abundance of the genus Bacteroides obtained from metagenomic analysis. Stool consistency was assessed using the Bristol Stool Scale, recoded so that higher values indicate more optimal stool form. Standardized regression coefficients (β) are displayed along the paths. The model was estimated using Unweighted Least Squares due to non-normal data distribution. Model fit indices are reported in Table 1. The model explains 19% of the variance in psychological distress (R^2^ = 0.19).

After establishing the confirmatory model grouping vitamins and fiber, we examined its predictive power regarding psychological distress, considering the roles of *Bacteroides* abundance and stool consistency. Figure 2 shows the model estimated using Unweighted Least Squares due to the violations of multivariate normality (Bollen-Stine bootstrap p < 0.005) (Bollen and Stine, 1993). Table 1 presents the model’s goodness-of-fit indices, indicating an acceptable fit.

According to Cohen’s criteria (1992), regression coefficients are classified as small (*β* > = 0.14), medium (β > = 0.36), and large (β > = 0.51). The model demonstrated that Vitamins + Fiber and stool consistency directly predicted psychological distress (β = −0.38, β = −0.14, respectively), with higher Vitamins + Fiber levels and improved stool consistency associated with decreased distress.

Additionally, Vitamins + Fiber indirectly predicted distress mediated through *Bacteroides* abundance and stool consistency: Vitamins + Fiber decreased *Bacteroides* abundance (β = −0.17), higher *Bacteroides* abundance was associated with worse stool consistency (β = −0.80), and better stool consistency was associated with reduced distress (β = −0.14). Together, these predictors explained 19% of the variance in psychological distress (R^2^ = 0.19).

Consequently, vitamins and fiber appear to predict psychological distress both directly and indirectly in patients with ALS, suggesting that a diet rich in these nutrients may reduce psychological distress in this population.

## 4 Discussion

Anxiety and depression represent significant comorbidities in patients with ALS, often exacerbating clinical symptoms such as respiratory dysfunction and functional disability (Vignola et al., 2008; Carvalho et al., 2016; Young et al., 2022). Understanding modifiable factors that influence psychological well-being in this population is therefore clinical imperative. Other variables associated with the pathogenesis of ALS itself should also be considered, as they could underlie psychological distress. Similarly, dietary intake has been identified as relevant factor in disease progression (Genton et al., 2011), with clear nutritional alterations recently observed in our laboratory in a population sample of ALS similar to that of the current study (Carrera-Juliá et al., 2024).

In accordance with our main objective, our findings provide new evidence that the dietary intake of specific B vitamins (B1, B2, B9) and vitamin C, combined with adequate fiber consumption, may serve as protective factors against psychological distress in patients with ALS. This relationship appears to operate through both direct neurobiological mechanisms and indirect pathways involving gut microbiota composition and gastrointestinal function.

To analyze this objective in depth, we examined the factor structure of micronutrient intake to determine whether a latent factor emerged to group these measures based on their common variance. In this regard, the identification obtained of a unified nutritional factor combining these specific vitamins and fiber, reflects their common dietary sources and synergistic biological functions. Fruits, vegetables, legumes, and whole grains are primary sources of these nutrients (Neufingerl and Eilander, 2021), and previous research has demonstrated that antioxidant nutrients and fiber-rich foods are associated with better functional outcomes in patients with ALS at diagnosis (Nieves et al., 2016). Importantly, these benefits appear to result from the synergistic effects of combined micronutrients rather than individual nutrients. Fruits and vegetables provide optimal sources of these micronutrients, and their combination with low energy density and high fiber content may enhance therapeutic benefits (Azzolina et al., 2020).

Furthermore, our subsequent objective was to develop a predictive model to assess whether the presence of a specific intestinal bacterium and stool consistency mediate the effect of micronutrient intake on psychological distress (anxiety and depression). In this context, it is known that total dietary fiber intake is associated with a 10% lower likelihood of depression in adults and 57% lower likelihood in adolescents (Saghafian et al., 2023). Similarly, vitamins play an important role in improving the perception of anxiety and depression. Low plasma vitamin C levels corresponding to moderate or severe deficiency are associated with a higher prevalence of depressive symptoms and cognitive impairment risk in healthy individuals (Plevin and Galletly, 2020). Folic acid has a major impact on emotional state (Paul et al., 2004), and vitamin B1 and vitamin B2 supplements can help reduce anxiety and stress while improving sleep quality (Ghaleiha et al., 2016). These benefits are confirmed by our model, which directly demonstrates how vitamins B1, B2, B9, and C, together with fiber, reduce psychological distress (*β* = −0.38). However, our previous study indicated that intake of these vitamins was adequate and, in some cases, above recommended levels, while fiber intake was well below recommended levels (Carrera-Juliá et al., 2024). This highlights the pivotal role of fiber within these nutritional patterns.

The therapeutic potential of these nutrients likely stems from their complementary mechanisms of action. Dietary fiber serves as a prebiotic substrate that promotes beneficial microbial metabolism and strengthens intestinal barrier function (Khoshbin and Camilleri, 2020). Conversely, inadequate fiber intake can compromise mucosal integrity, as certain bacterial species degrade the protective mucus layer when preferred substrates are unavailable (Camilleri et al., 2019). This explains why adequate vitamin absorption and bioavailability may depend on sufficient fiber intake, even when vitamin consumption appears adequate.

A diet rich in fiber and micronutrients (such as B vitamins) can positively modulate gut microbiota, reduce endotoxemia and neuroinflammation, and improve cognitive health and emotional well-being (Randeni and Xu, 2025). Śliwiński and Gawlik-Kotelnicka (2024) describe how vitamins B1, B2, B6, B9, and B12 improve depression by reducing oxidative stress, inflammation, and intestinal permeability, while also regulating the microbiota. The presence of intestinal dysbiosis, especially involving bacteria from the *Bacteroides* genus bacteria, in low vitamin and fiber diets (Wu et al., 2011; Calatayud et al., 2021; Aljuraiban et al., 2023) is associated with mental disorders such as anxiety and depression (Mason et al., 2020; Knuesel and Mohajeri, 2021; Liu et al., 2023; Zhao et al., 2024) due to changes in tryptophan metabolism and neurotransmitter production (Zhang et al., 2022). Our model demonstrates that inadequate nutrient intake indirectly influences anxiety and depression through gastrointestinal symptoms, stemming from elevated *Bacteroides* levels among the hundreds of bacteria analyzed. Specifically, *Bacteroides* was the only genus that exhibited a correlation of at least | ± 0.10| with anxiety and depression. This criterion was established because, according to Cohen (1992), a correlation value of 0.10 represents the threshold for a small effect size. In this regard, it has been observed how increased *Bacteroides* presence promotes constipation (Maeta et al., 2024; Wang et al., 2025), and this *Bacteroides*-mediated constipation is accompanied by anxiety (Liang et al., 2022), as observed in our ALS population. Therefore, we consider our results to be particularly relevant. While the relationship between gastrointestinal symptoms such as constipation and emotional disorders like depression has been previously described in ALS (Niu et al., 2022), we have identified the predictors of these disorders using our model.

A plausible mechanistic explanation for these findings can be framed within the gut–brain axis, a bidirectional communication system integrating neural, immune, and metabolic signaling pathways. Recent studies have reinforced the role of gut microbiota in modulating brain function and behavior through multiple mechanisms, including the production of short-chain fatty acids (SCFAs), neurotransmitter precursors, and immune mediators (Schneider et al., 2024). These microbiota-derived signals can influence intestinal barrier integrity, systemic inflammation, and neuroinflammatory processes, all of which are particularly relevant in neurodegenerative conditions (Chen et al., 2025). Notably, emerging research highlights the role of neuroimmune pathways, including microglial activation, as key mediators linking gut microbiota alterations with central nervous system dysfunction (Keane et al., 2025). In parallel, diet-driven changes in microbiota composition can alter gastrointestinal motility and stool consistency, reflecting functional alterations in the gut environment. In the context of ALS, where systemic inflammation and neurodegeneration coexist, this complex interaction between dietary factors, microbiota composition, and gut function may contribute to psychological distress through immune, metabolic, and neural pathways, providing a biologically plausible framework for the associations observed in this study.

### 4.1 Limitations

Several limitations should be considered when interpreting these findings. First, the sample size (*n* = 48) was limited by the rarity of ALS, which may have reduced the statistical power to detect smaller effect sizes and could limit the generalizability of the results to broader ALS populations.

Second, the cross-sectional design precludes causal inferences regarding the temporal relationships between nutritional intake, microbiota composition, and psychological symptoms. Consequently, the observed directional trajectories within the model must be interpreted as statistical associations rather than evidence of causality. In this regard, longitudinal studies are needed to establish whether nutritional interventions can prospectively improve psychological outcomes and to examine how these relationships evolve alongside disease progression. Furthermore, when interpreting the statistical findings, it is essential to acknowledge that the reliance on self-reported dietary intake and psychological measures may introduce information bias and measurement errors, including potential recall bias.

Third, although our model explained 19% of the variance in psychological distress, other unmeasured factors—including disease severity, social support, medication effects, and genetic predisposition—likely contribute substantially to emotional well-being in this population. Future research should incorporate these variables to develop more comprehensive predictive models with higher explanatory power.

Fourth, the study population was limited to Spanish adults, which may limit generalizability to other ethnicities or geographic regions with distinct dietary patterns and genetic backgrounds. Additionally, the exclusion of patients with advanced disease (e.g., those requiring tracheostomy or ventilatory support) may have resulted in a sample with less severe ALS, potentially affecting the observed relationships.

### 4.2 Clinical implications and future direction

Clinically, these findings support the implementation of routine nutritional screening to ensure B vitamin and fiber adequacy within ALS care protocols. The specific focus on *Bacteroides* abundance as a potential biomarker warrants further investigation through intervention studies. Future research should examine whether structured nutritional counseling or supplementation targeting these specific nutrients can prospectively improve psychological outcomes and quality of life in patients with ALS.

### 4.3 Significance

This study is novel because it establishes a predictive model demonstrating that the intake of a nutritional factor comprising vitamins B1, B2, B9, C, and fiber is inversely associated with psychological distress. This relationship occurs both directly and indirectly, mediated by a lower abundance of *Bacteroides* bacteria and improved stool consistency in patients with ALS.

## 5 Conclusion

The integration of nutritional, microbiological, and psychological assessments represents a promising direction for personalized ALS care, potentially offering patients and families additional tools for symptom management and improved quality of life in addition to standard neurological treatments. Specifically, the intake of vitamins B1, B2, B9, and C, in conjunction with fiber, appears to play a pivotal role in emotional well-being. This effect occurs, not only directly but also indirectly through the mitigation of gastrointestinal symptoms mediated by *Bacteroides* presence in the gut microbiota.

## Data Availability

The raw data generated in this study can be found here: https://www.ebi.ac.uk/ena, accession PRJEB90512.

## Footnotes

1
http://cmprod1.cibio.unitn.it/biobakery4/metaphlan_databases/

2
https://dietopro.com/
