# Supplementary material for: Interplay between B vitamins, fiber, and Bacteroides abundance: a predictive model for anxiety and depression in amyotrophic lateral sclerosis
Source: Front Microbiol. 2026 Jun 8;17:1815390. doi: 10.3389/fmicb.2026.1815390 (PMC13283967; doi:10.3389/fmicb.2026.1815390)
Supplement: Supplementary file 1 [file Table_1.DOCX]

**SUPPLEMENTARY TABLE 1**

Descriptive statistics and Pearson correlations of the measures

| **Measures** | **1** | **2** | **3** | **4** | **5** | **6** | **7** | **8** | **9** | **10** | **11** | **12** | **13** | **14** | **15** | **16** | **17** | **18** | **19** | **20** | **21** | **22** | **23** | **24** | **25** | **26** |
| --- | --- | --- | --- | --- | --- | --- | --- | --- | --- | --- | --- | --- | --- | --- | --- | --- | --- | --- | --- | --- | --- | --- | --- | --- | --- | --- |
| 1. Sodium (g) | 1.00 |  |  |  |  |  |  |  |  |  |  |  |  |  |  |  |  |  |  |  |  |  |  |  |  |  |
| 1. Fiber (g) | 0.06 | 1.00 |  |  |  |  |  |  |  |  |  |  |  |  |  |  |  |  |  |  |  |  |  |  |  |  |
| 1. Ethanol (g) | -0.24 | 0.29 | 1.00 |  |  |  |  |  |  |  |  |  |  |  |  |  |  |  |  |  |  |  |  |  |  |  |
| 1. Iodine (µg) | 0.05 | 0.80 | 0.60 | 1.00 |  |  |  |  |  |  |  |  |  |  |  |  |  |  |  |  |  |  |  |  |  |  |
| 1. Potassium (mg) | 0.49 | 0.22 | -0.20 | 0.17 | 1.00 |  |  |  |  |  |  |  |  |  |  |  |  |  |  |  |  |  |  |  |  |  |
| 1. Calcium (mg) | 0.24 | 0.28 | 0.16 | 0.47 | 0.44 | 1.00 |  |  |  |  |  |  |  |  |  |  |  |  |  |  |  |  |  |  |  |  |
| 1. Magnesium (mg) | 0.41 | 0.51 | -0.20 | 0.44 | 0.63 | 0.58 | 1.00 |  |  |  |  |  |  |  |  |  |  |  |  |  |  |  |  |  |  |  |
| 1. Phosphorus (mg) | 0.42 | 0.32 | -0.21 | 0.39 | 0.54 | 0.77 | 0.79 | 1.00 |  |  |  |  |  |  |  |  |  |  |  |  |  |  |  |  |  |  |
| 1. Iron (mg) | 0.09 | 0.94 | 0.04 | 0.68 | 0.19 | 0.19 | 0.52 | 0.35 | 1.00 |  |  |  |  |  |  |  |  |  |  |  |  |  |  |  |  |  |
| 1. Selenium (µg) | 0.29 | 0.82 | -0.08 | 0.68 | 0.36 | 0.39 | 0.64 | 0.58 | 0.88 | 1.00 |  |  |  |  |  |  |  |  |  |  |  |  |  |  |  |  |
| 1. Zinc (mg) | 0.11 | 0.95 | 0.03 | 0.69 | 0.22 | 0.24 | 0.54 | 0.37 | 0.98 | 0.89 | 1.00 |  |  |  |  |  |  |  |  |  |  |  |  |  |  |  |
| 1. Vitamin B1 (mg) | 0.09 | 0.96 | 0.06 | 0.68 | 0.20 | 0.19 | 0.50 | 0.33 | 0.98 | 0.87 | 0.99 | 1.00 |  |  |  |  |  |  |  |  |  |  |  |  |  |  |
| 1. Vitamin B2 (mg) | 0.10 | 0.96 | 0.07 | 0.69 | 0.20 | 0.20 | 0.51 | 0.34 | 0.98 | 0.88 | 0.99 | 1.00 | 1.00 |  |  |  |  |  |  |  |  |  |  |  |  |  |
| 1. Vitamin B6 (mg) | -0.10 | 0.87 | 0.71 | 0.88 | 0.00 | 0.24 | 0.21 | 0.08 | 0.72 | 0.56 | 0.72 | 0.74 | 0.75 | 1.00 |  |  |  |  |  |  |  |  |  |  |  |  |
| 1. Vitamin B12 (µg) | 0.08 | 0.97 | 0.13 | 0.74 | 0.18 | 0.23 | 0.51 | 0.34 | 0.98 | 0.88 | 0.98 | 0.99 | 0.99 | 0.78 | 1.00 |  |  |  |  |  |  |  |  |  |  |  |
| 1. Folate B9 (µg) | 0.37 | 0.66 | 0.06 | 0.56 | 0.44 | 0.62 | 0.75 | 0.61 | 0.58 | 0.65 | 0.60 | 0.59 | 0.59 | 0.45 | 0.60 | 1.00 |  |  |  |  |  |  |  |  |  |  |
| 1. Niacin B3 (mg) | -0.23 | 0.39 | 0.99 | 0.67 | -0.16 | 0.19 | -0.14 | -0.17 | 0.15 | 0.02 | 0.14 | 0.17 | 0.17 | 0.78 | 0.23 | 0.14 | 1.00 |  |  |  |  |  |  |  |  |  |
| 1. Vitamin C (mg) | 0.23 | 0.45 | -0.17 | 0.30 | 0.38 | 0.42 | 0.60 | 0.39 | 0.43 | 0.51 | 0.48 | 0.43 | 0.43 | 0.20 | 0.44 | 0.77 | -0.10 | 1.00 |  |  |  |  |  |  |  |  |
| 1. Vitamin A (µg) | 0.15 | 0.17 | -0.15 | 0.19 | 0.32 | 0.17 | 0.41 | 0.28 | 0.16 | 0.23 | 0.17 | 0.14 | 0.14 | 0.00 | 0.18 | 0.49 | -0.12 | 0.59 | 1.00 |  |  |  |  |  |  |  |
| 1. Vitamin D (µg) | 0.13 | 0.93 | 0.03 | 0.71 | 0.21 | 0.28 | 0.53 | 0.41 | 0.97 | 0.91 | 0.98 | 0.98 | 0.99 | 0.72 | 0.99 | 0.60 | 0.14 | 0.45 | 0.16 | 1.00 |  |  |  |  |  |  |
| 1. Vitamin E (mg) | 0.13 | 0.95 | 0.02 | 0.68 | 0.24 | 0.25 | 0.57 | 0.39 | 0.98 | 0.89 | 0.99 | 0.99 | 0.99 | 0.71 | 0.98 | 0.65 | 0.13 | 0.51 | 0.20 | 0.98 | 1.00 |  |  |  |  |  |
| 1. Stool Type (Bristol) | -0.18 | 0.07 | -0.16 | -0.04 | 0.03 | 0.00 | 0.16 | 0.01 | 0.09 | 0.10 | 0.09 | 0.09 | 0.09 | -0.04 | 0.11 | 0.16 | -0.15 | 0.25 | 0.14 | 0.10 | 0.09 | 1.00 |  |  |  |  |
| 1. *Bacteroides* | -0.08 | -0.15 | 0.03 | -0.12 | 0.02 | -0.13 | -0.17 | -0.16 | -0.19 | -0.23 | -0.17 | -0.14 | -0.14 | -0.08 | -0.15 | -0.14 | 0.00 | -0.19 | -0.14 | -0.15 | -0.15 | -0.29 | 1.00 |  |  |  |
| 1. Depression (ADI-12) | -0.19 | -0.27 | -0.20 | -0.20 | -0.10 | -0.19 | -0.17 | -0.12 | -0.17 | -0.14 | -0.19 | -0.18 | -0.19 | -0.27 | -0.20 | -0.35 | -0.23 | -0.28 | -0.24 | -0.19 | -0.20 | -0.04 | 0.11 | 1.00 |  |  |
| 1. Somatic Anxiety (BAI) | -0.18 | -0.22 | -0.17 | -0.31 | 0.05 | -0.14 | -0.23 | -0.13 | -0.15 | -0.18 | -0.17 | -0.15 | -0.16 | -0.22 | -0.18 | -0.34 | -0.18 | -0.35 | -0.24 | -0.18 | -0.17 | -0.20 | 0.28 | 0.62 | 1.00 |  |
| 1. Cognitive Affective Anxiety (BAI) | -0.06 | -0.08 | -0.14 | -0.24 | 0.05 | -0.24 | -0.22 | -0.19 | 0.01 | -0.10 | -0.02 | 0.01 | 0.00 | -0.08 | -0.04 | -0.33 | -0.13 | -0.37 | -0.29 | -0.04 | -0.03 | -0.23 | 0.22 | 0.51 | 0.90 | 1.00 |
| - *M* | 3806.71 | 30.9 | 91.66 | 218.6 | 4087.13 | 1023.27 | 398.41 | 1553.77 | 26.93 | 127.96 | 18.68 | 7.99 | 9.08 | 14.26 | 16.04 | 318.03 | 75.49 | 174.55 | 1133.13 | 14.59 | 20.04 | 2.98 | 1.13 | 10.03 | 20.90 | 18.70 |
| - *SD* | 10895.6 | 53.13 | 621.41 | 246.08 | 2323.34 | 519.36 | 223.9 | 726.83 | 63.93 | 105.18 | 49.76 | 48.33 | 48.55 | 64.3 | 54.83 | 157.51 | 277.95 | 114.23 | 1013.89 | 48.10 | 50.02 | 1.73 | 0.90 | 7.58 | 7.50 | 7.74 |

**Note:** Correlations ≥ |±0.29| are statistically significant.
